# Supplementary material for: Heterogeneous RNA editing and influence of ADAR2 on mesothelioma chemoresistance and the tumor microenvironment
Source: Mol Oncol. 2022 Oct 31;16(22):3949–74. doi: 10.1002/1878-0261.13322 (PMC9718120; doi:10.1002/1878-0261.13322)
Supplement: Supplementary file 1 — Fig. S1. A to G RNA editing is correlated with ADAR1 and ADAR2 mRNA and varies with splicing inhibition. Fig. S2. Splicing is correlated to A‐to‐I RNA editing levels. Fig. S3. Characteristics of ADAR1 and ADAR2 expression in mesothelioma. Fig. S4. ADAR2 expression correlates with BAP1 status but not with clinical outcome. Fig. S5. Expression of empty vector does not rescue COPA editing in Adar2‐deficient cells. Fig. S6. (A) RT‐PCR validation of FLNB exon 30 alternate splicing in Mero95 WT, KD, and Rescue spheroids on 2% agarose gel. Bands were quantified using imagej software. The ratio of exclusion band to the sum of exclusion and inclusion band relative to WT is represented as Relative FLNB exon30 skipping. n = 3. One‐way ANOVA with post‐hoc Tukey's test. Error bars indicate SEM. (B) ADAR2 protein expression is not associated with response to cisplatin/pemetrexed. Comparison of ADAR2 immunoreactivity and response in patients treated with pemetrexed/cisplatin induction chemotherapy. Fig. S7. Silencing of TMEM173 encoding STING is more effective in downregulating ISG expression in Mero95 ADAR2 KD cells, compared to MAVS silencing. Fig. S8. ADAR2 deficiency leads to changes in the tumor microenvironment in vivo. Fig. S9. Contribution of RNA editing to mesothelioma heterogeneity. [file MOL2-16-3949-s001.docx]

**Supplementary Figures**


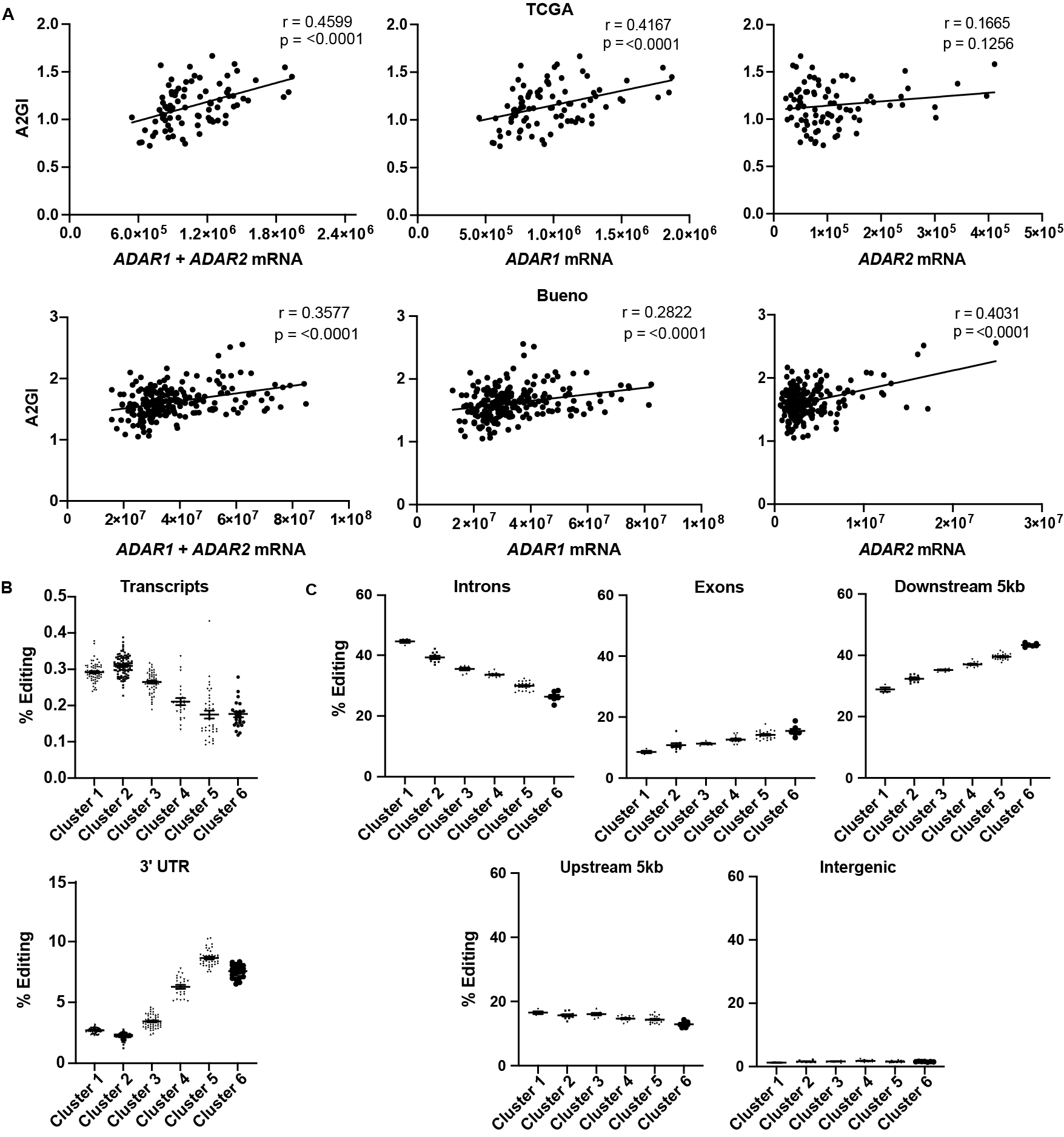


**Figure S1**. **A to G RNA editing is correlated with *ADAR1* and *ADAR2* mRNA and varies with splicing inhibition** (A) A2GI values are plotted against the sum of *ADAR1* and *ADAR2* mRNA and against *ADAR1* and *ADAR2* mRNA levels in TCGA (n=86) and Bueno (n=211) data sets. Pearson correlation and linear regression analysis. (B) Contribution of transcripts and 3’UTR editing to the cluster profile of exons in Figure 1D. (n=297, Cluster 1 – 63, Cluster 2 – 95, Cluster 3 – 51, Cluster 4 – 25, Cluster 5 – 39, Cluster 6 – 24) Error bars indicate SEM. (C) Unsupervised clustering of editing in primary mesothelioma cultures (FunGeST series) defined 6 groups. (n=64, Cluster 1 – 4, Cluster 2 – 10, Cluster 3 – 7, Cluster 4 – 13, Cluster 5 – 23, Cluster 6 – 7) Graphs represent the percentage of A-to-I RNA editing within the specified genomic region within each cluster. Significance is shown in Table S5. Error bars indicate SEM.


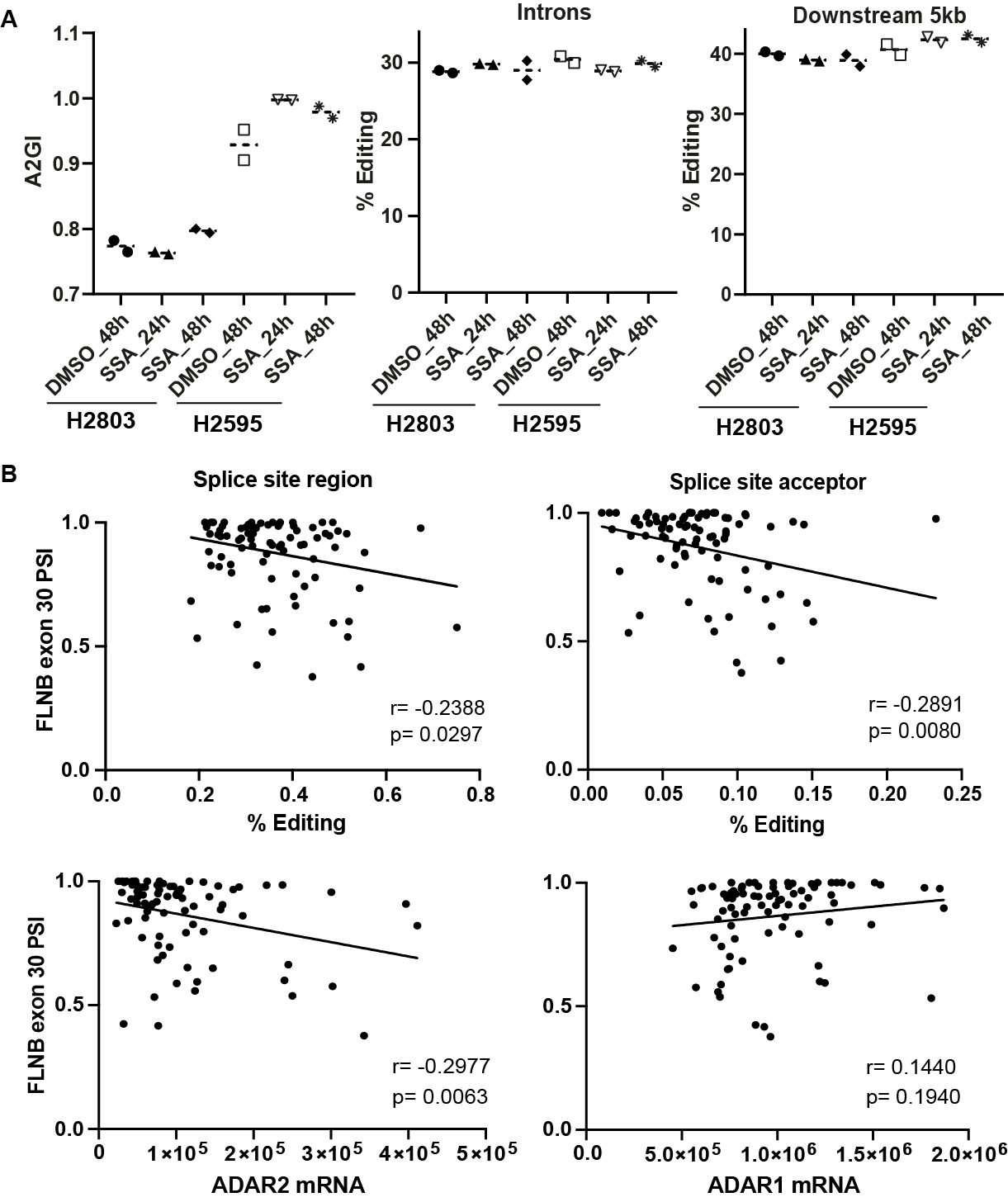


**Fig S2. Splicing is correlated to A-to-I RNA editing levels**. (A) RNA-seq data on mesothelioma cell lines NCI-H2803 and NCI-H2595 treated with spliceosome inhibitor SSA or DMSO (for 24 or 48 hours) (1) were used to compute A to G editing and the percentage of editing levels in introns and regions 5kb downstream of genes. n=2. (B) Percentage spliced in (PSI) of FLNB exon 30 in comparison to editing at splice site (upper panel) and ADARs expression (lower panel). n=83. Pearson correlation and linear regression analysis.


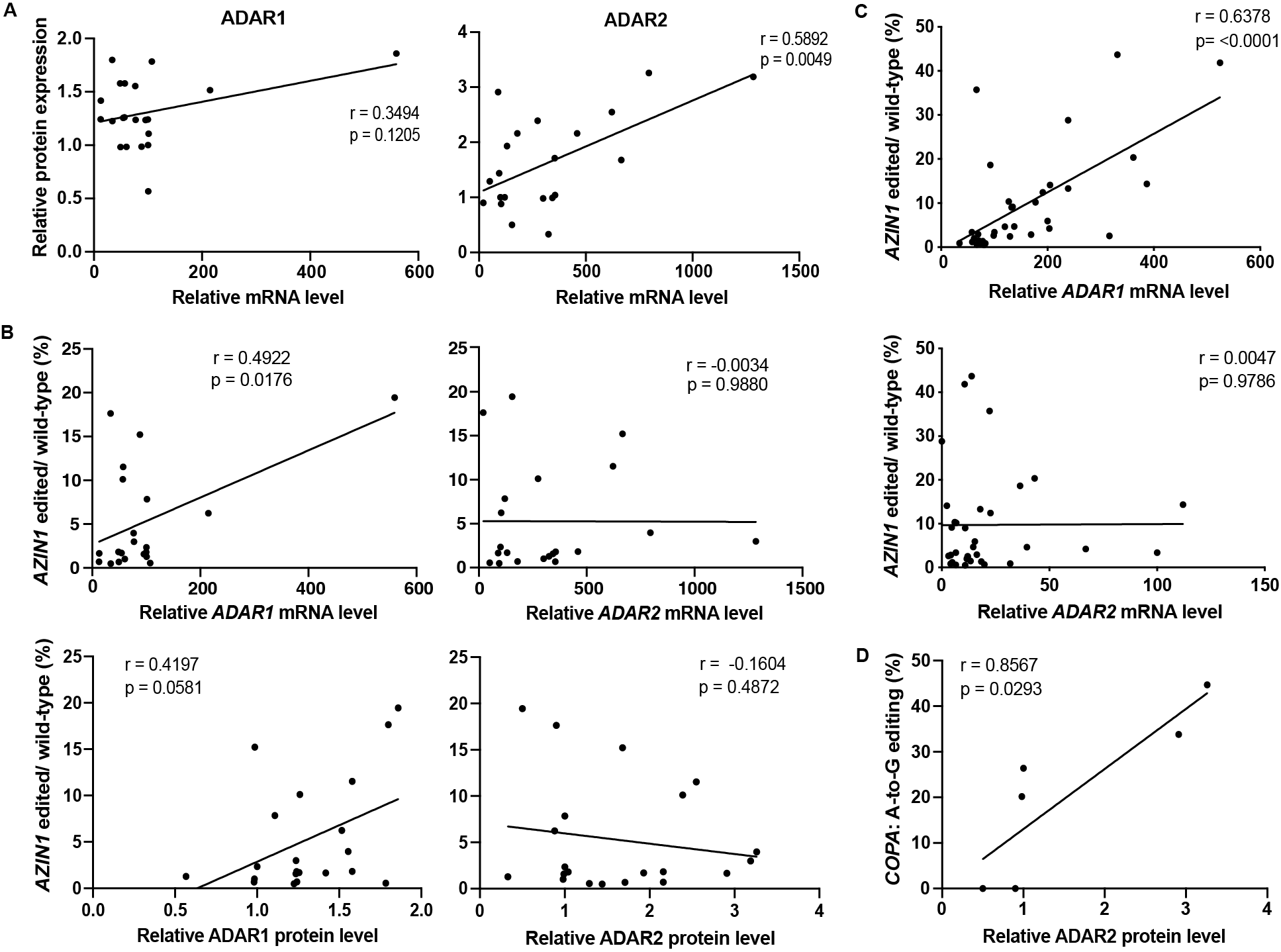


**Figure S3.** **Characteristics of** **ADAR1 and ADAR2 expression in mesothelioma.** (A) Relative ADAR1 and ADAR2 protein levels are plotted against their respective relative mRNA levels in the cell lines; each dot represents one cell line (n=21). Pearson correlation and linear regression analysis. (B) *AZIN1* editing levels in the cell lines (n=21) are plotted against relative *ADAR1* and *ADAR2* mRNA or protein levels. Pearson correlation and linear regression analysis. (C) *AZIN1* editing levels in primary mesothelioma cultures (n=34) are plotted against relative *ADAR1* and *ADAR2* mRNA levels. Pearson correlation and linear regression analysis. (D) Editing levels of *COPA* are plotted against the relative ADAR2 protein levels in SDM104, ACC Meso1, SPC111, SPC212, ONE58 and Mero95 cell lines. Pearson correlation and linear regression analysis.


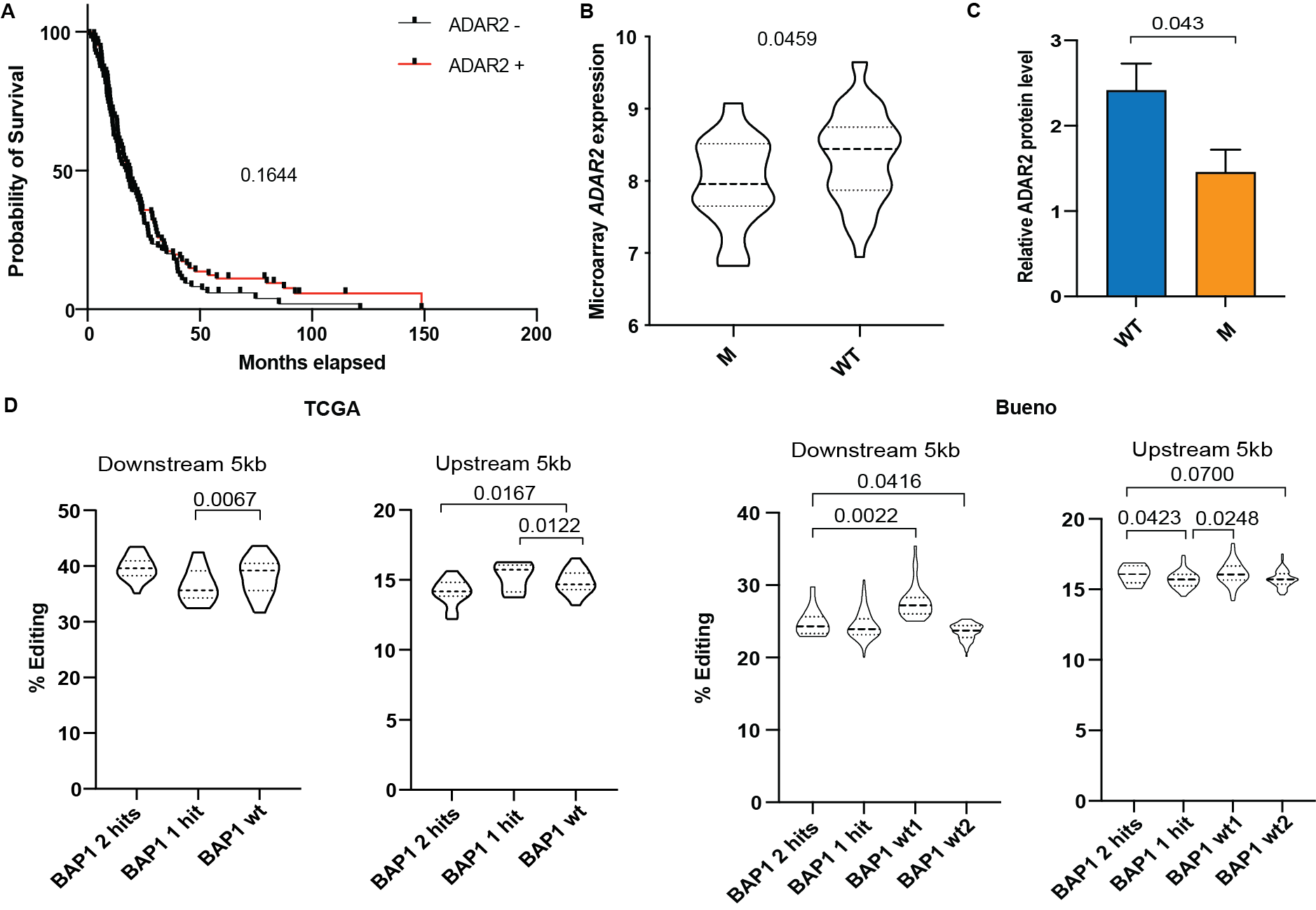


**Figure S4.** **ADAR2 expression correlates with BAP1 status but not with clinical outcome** (A) Kaplan-Meier Survival curve for patients with ADAR2 positive or negative staining in TMA. (B) Expression level of *ADAR2* in the pleural mesothelioma CIT series (2, 3) based on the status of BAP1 (M – mutated (n=16), WT – wild-type (n=44)). Violin plots show the median and quartiles. Unpaired t-test. (C) Relative expression of ADAR2 protein level in mesothelioma cell lines based on BAP1 status (WT -wild-type (n=7), M – mutated (n=6)). Unpaired t-test. Error bars indicate SEM. (D) A-to-I RNA editing levels in regions 5kb downstream and 5kb upstream of genes from TCGA and Bueno datasets according to the BAP1 status (wt- wild type BAP1 (TCGA, n=25; Bueno, n=126), mutated BAP1 - 1 hit (TCGA, n=10; Bueno, n=61) or 2 hit (TCGA, n=18; Bueno, n=13)). Violin plots show the median and quartiles. Mann-Whitney test.


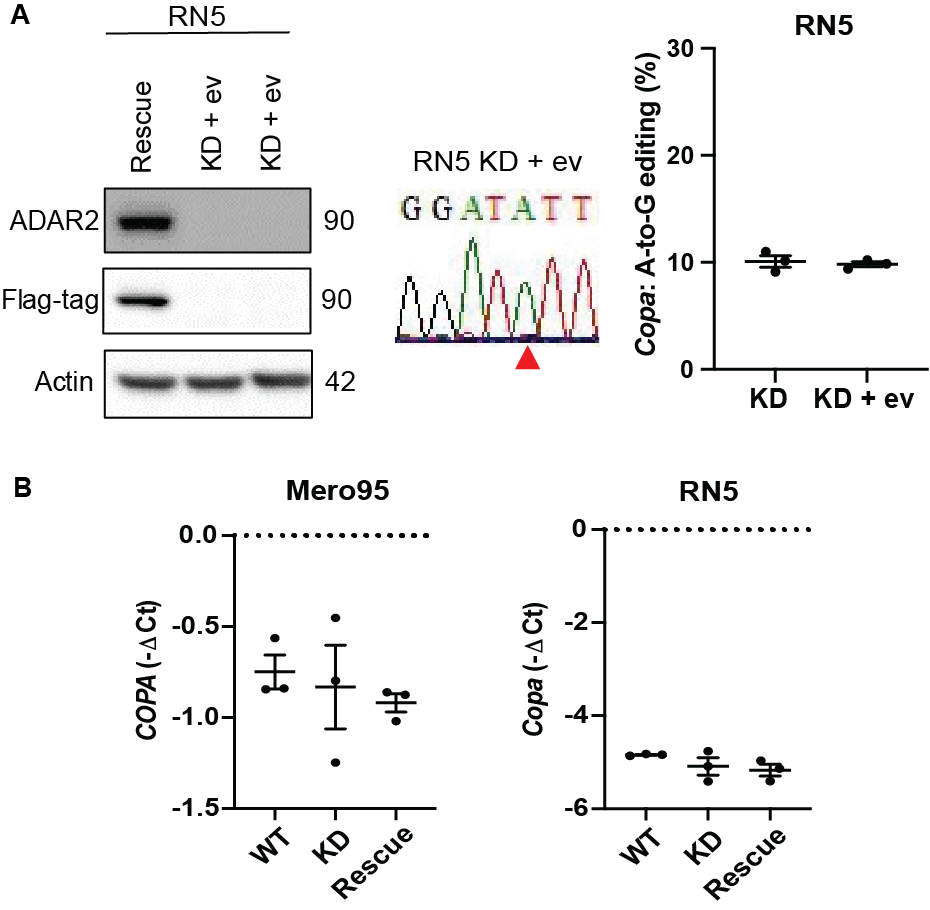


**Figure S5.** **Expression of empty vector does not rescue *COPA* editing in Adar2 deficient cells**. (A) Adar2 protein expression (left) in RN5 Adar2 rescue cells compared to the expression in two different RN5 Adar2 KD clones transfected with empty vector (ev) and selected with blasticidine. Sequence chromatogram (middle) of *Copa* transcript from RN5 KD cells transfected with empty vector. Red arrow indicates the Ile/Val editing position. Quantification of A-to-G changes (right) in *Copa* cDNA from sequence chromatogram, comparing RN5 KD cells to RN5 KD cells transfected with empty vector (KD+ev). n=3. Paired t-test. Error bars indicate SEM. (B) Relative mRNA expression of *COPA* in Mero95 and RN5 WT, KD and rescue cells. n=3. One-Way ANOVA. Error bars indicate SEM.


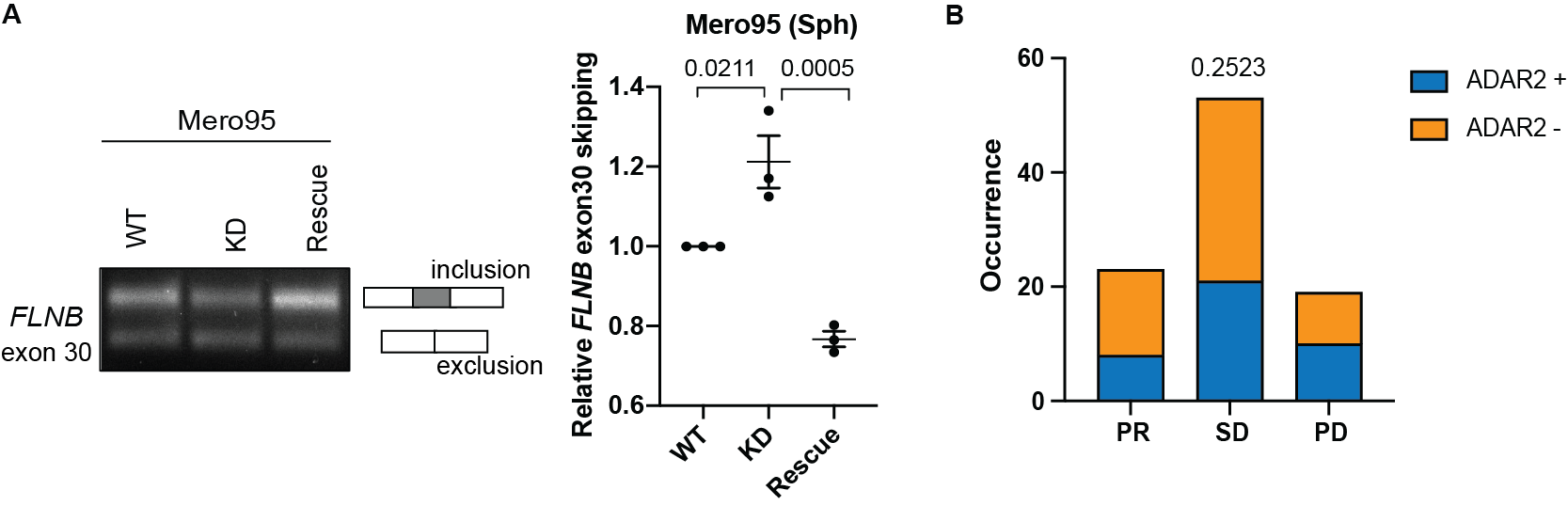


**Figure S6.** (A) RT-PCR validation of *FLNB* exon 30 alternate splicing in Mero95 WT, KD, and Rescue spheroids on 2% agarose gel. Bands were quantified using ImageJ software. The ratio of exclusion band to the sum of exclusion and inclusion band relative to WT is represented as Relative *FLNB* exon30 skipping. n=3. One-way ANOVA with post-hoc Tukey’s test. Error bars indicate SEM. (B) ADAR2 protein expression is not associated with response to cisplatin/pemetrexed. Comparison of ADAR2 immunoreactivity and response in patients treated with pemetrexed/ cisplatin induction chemotherapy. PR: partial responder . SD: stable disease. PD: progressive disease. Chi-square test for trend.


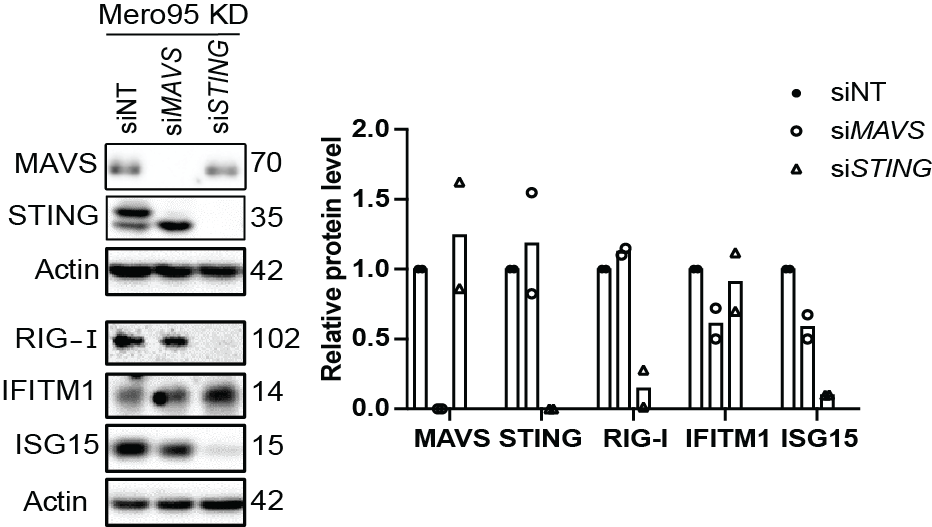


**Figure S7.** **Silencing of *TMEM173* encoding STING is more effective in downregulating ISG expression in Mero95 ADAR2 KD cells, compared to *MAVS* silencing**. Expression of ISGs (RIG-I, IFITM1 and ISG15) upon silencing of either *MAVS* or *STING* for 96 hours in Mero95 ADAR2 KD cells. Protein levels are represented relative to siNT. Protein size is represented in kDa. (n=2)

**
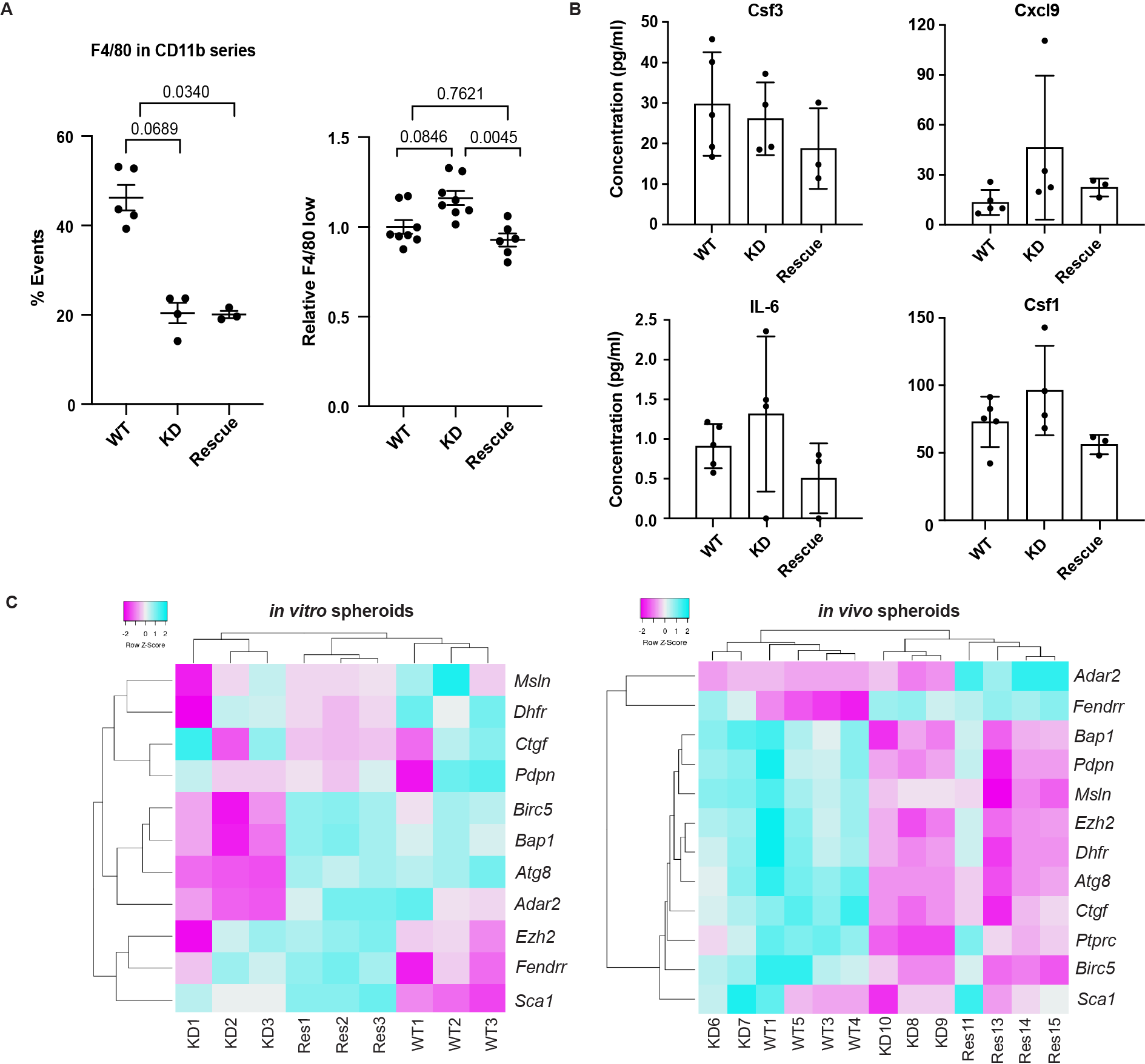
Figure S8. ADAR2 deficiency leads to changes in the tumor microenvironment *in vivo*.** RN5 WT, KD and rescue cells were injected i.p. into syngeneic mice and two weeks later the animals were sacrificed and peritoneal lavage was collected to analyze (A) Cd11b^+^F4/80 population and relative F4/80low in Cd45^+^ series and Cd11b^+^ series, WT, n=5, KD, n=4, Rescue, n=3, Kruskal-Wallis test, Error bars indicate SEM (B) cytokines/chemokines, One-way ANOVA with post-hoc Tukey’s test, Error bars indicate SEM. (C) Spheroids were then collected to extract RNA and the expression of tumor-associated genes (normalized to beta-actin) was used to create a heatmap (right, WT, n=4, KD, n=5, Rescue, n=4) which was compared to expression *in vitro* (left, n=3). Cd45 (encoded by *Ptprc*) was used to show variability of hematopoietic compartment in the *in vivo* settings.


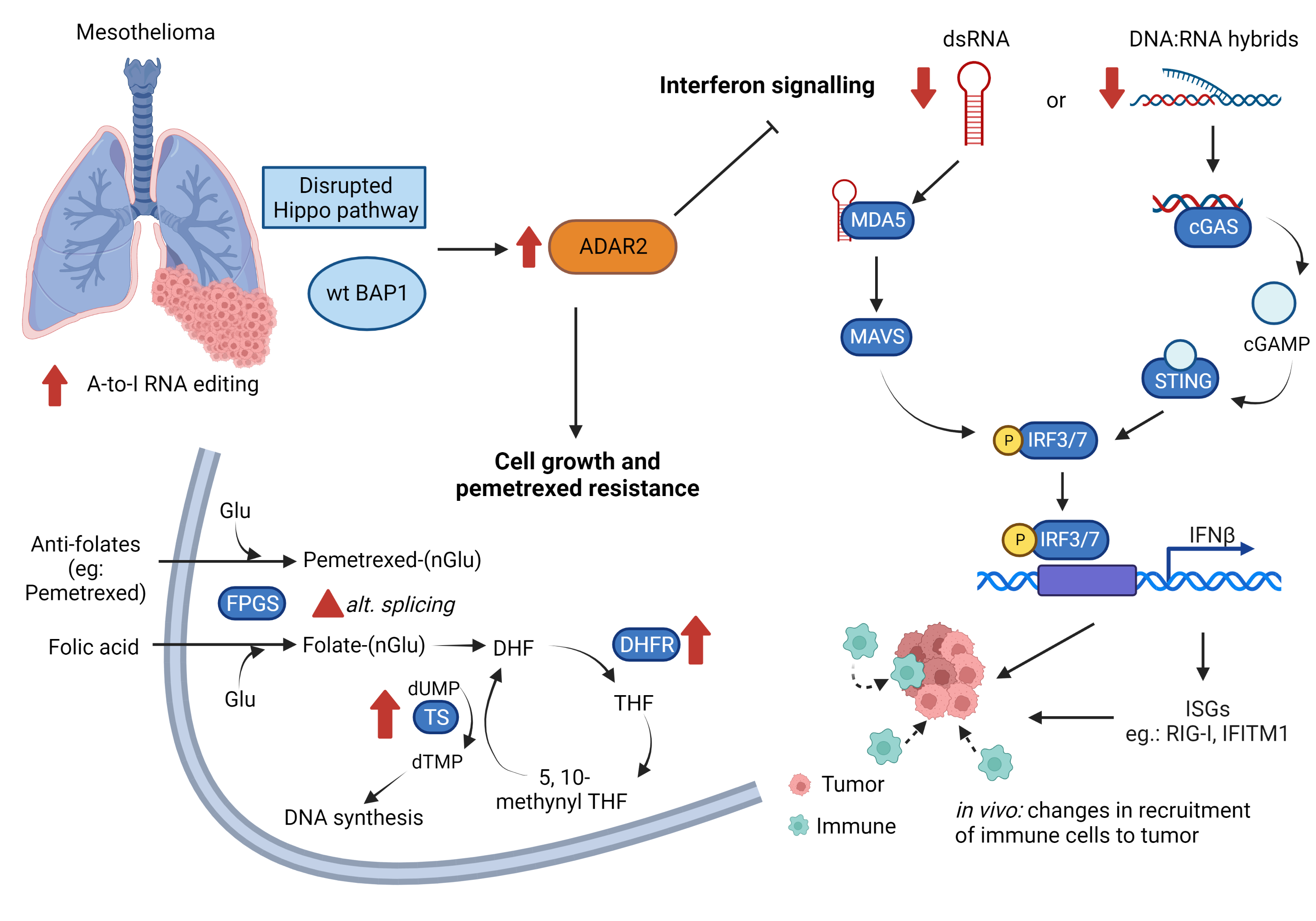


**Figure S9.** **Contribution of RNA editing to mesothelioma heterogeneity.** Higher levels of A-to-I RNA editing have been observed in human mesothelioma compared to normal mesothelial tissue. Disrupted Hippo pathway and wild-type BAP1 are associated with increased levels of RNA editing enzyme ADAR2.. Increased levels of ADAR2 results in a) increased cell growth and pemetrexed resistance, via upregulated expression of pemetrexed targets, DHFR and TS, and changes in alternate splicing of FPGS, and b) the blockade of interferon signaling, downstream of the destabilization of dsRNA and DNA:RNA hybrid structures, which would otherwise activate interferon signaling via MDA5/MAVS or STING-cGAS pathways, respectively. Created with BioRender.com

References

1. Bueno R, Stawiski EW, Goldstein LD, Durinck S, De Rienzo A, Modrusan Z, et al. Comprehensive genomic analysis of malignant pleural mesothelioma identifies recurrent mutations, gene fusions and splicing alterations. Nat Genet. 2016;48(4):407-16.

2. Quetel L, Meiller C, Assie JB, Blum Y, Imbeaud S, Montagne F, et al. Genetic alterations of malignant pleural mesothelioma: association to tumor heterogeneity and overall survival. Mol Oncol. 2020.

3. Blum Y, Meiller C, Quetel L, Elarouci N, Ayadi M, Tashtanbaeva D, et al. Dissecting heterogeneity in malignant pleural mesothelioma through histo-molecular gradients for clinical applications. Nat Commun. 2019;10(1):1333.
